# Delineation of 12-Lead ECG Representative Beats Using Convolutional Encoder–Decoders with Residual and Recurrent Connections

**DOI:** 10.3390/s24144645

**Published:** 2024-07-17

**Authors:** Vessela Krasteva, Todor Stoyanov, Ramun Schmid, Irena Jekova

**Affiliations:** 1 Institute of Biophysics and Biomedical Engineering, Bulgarian Academy of Sciences, Acad. G. Bonchev Str. Bl. 105, 1113 Sofia, Bulgaria; vessika@biomed.bas.bg (V.K.); todor@biomed.bas.bg (T.S.); 2 Signal Processing, Schiller AG, Altgasse 68, CH-6341 Baar, Switzerland; ramun.schmid@schiller.ch

**Keywords:** deep learning, deep neural networks, ECG signal processing, ECG segmentation, average beat, ECG interval measurements, diagnostic ECG waves

## Abstract

The aim of this study is to address the challenge of 12-lead ECG delineation by different encoder–decoder architectures of deep neural networks (DNNs). This study compares four concepts for encoder–decoders based on a fully convolutional architecture (CED-Net) and its modifications with a recurrent layer (CED-LSTM-Net), residual connections between symmetrical encoder and decoder feature maps (CED-U-Net), and sequential residual blocks (CED-Res-Net). All DNNs transform 12-lead representative beats to three diagnostic ECG intervals (P-wave, QRS-complex, QT-interval) used for the global delineation of the representative beat (P-onset, P-offset, QRS-onset, QRS-offset, T-offset). All DNNs were trained and optimized using the large PhysioNet ECG database (PTB-XL) under identical conditions, applying an advanced approach for machine-based supervised learning with a reference algorithm for ECG delineation (ETM, Schiller AG, Baar, Switzerland). The test results indicate that all DNN architectures are equally capable of reproducing the reference delineation algorithm’s measurements in the diagnostic PTB database with an average P-wave detection accuracy (96.6%) and time and duration errors: mean values (−2.6 to 2.4 ms) and standard deviations (2.9 to 11.4 ms). The validation according to the standard-based evaluation practices of diagnostic electrocardiographs with the CSE database outlines a CED-Net model, which measures P-duration (2.6 ± 11.0 ms), PQ-interval (0.9 ± 5.8 ms), QRS-duration (−2.4 ± 5.4 ms), and QT-interval (−0.7 ± 10.3 ms), which meet all standard tolerances. Noise tests with high-frequency, low-frequency, and power-line frequency noise (50/60 Hz) confirm that CED-Net, CED-Res-Net, and CED-LSTM-Net are robust to all types of noise, mostly presenting a mean duration error < 2.5 ms when compared to measurements without noise. Reduced noise immunity is observed for the U-net architecture. Comparative analysis with other published studies scores this research within the lower range of time errors, highlighting its competitive performance.

## 1. Introduction

The electrocardiogram (ECG) is a standard clinical tool for the investigation of the heart’s electrical activity. The technique is easily accessible and cost-effective, related to non-invasive ECG signal recording by means of skin surface electrodes connected to an electronic measurement device (electrocardiograph) [1]. The ECG bio-signal is produced by depolarization and repolarization currents flowing in different parts of the myocardium [2]. These currents are triggered by synchronized events within the cardiac cycle and define the standard patterns seen in the ECG, including the following: P-wave (atrial depolarization) initiating atrial contraction (systole); QRS-complex (ventricular depolarization) initiating ventricular systole; T-wave (ventricular repolarization) marking the beginning of ventricular relaxation; and an isoelectric line (resting membrane potentials). The position of the electrodes on the body surface determines the angle or lead to viewing the electrical vector of the heart, which can generally be interpreted in the three-dimensional space of the frontal, horizontal, and sagittal planes [3]. The information obtained from the standard 12-lead ECG, including six limb leads (I, II, III, avR, avL, avF) and six chest leads (V1, V2, V3, V4, V5, V6), is, however, considered as a gold standard for the diagnosis of cardiovascular pathologies [4,5]. Global measurements are made on ECG waveform templates constructed for each lead from dominant complexes in the recording. Clinically useful information is mainly derived from ECG intervals and amplitudes belonging to P-, QRS-, and T-waves, defined by several fiducial points (characteristic wave peaks, onset and offset boundaries). Their measurement, also referred to in the literature as ECG delineation or segmentation, can be made from individual lead data or from mathematical combinations of simultaneously acquired individual lead data [6]. Measurement error has an important effect on the accuracy of ECG diagnostic statements [7]; therefore, standards recommend that ECG delineation systems provide their error tolerance limits obtained on precisely annotated test sets [8]. The development of accurate and robust methods for automatic ECG delineation has been a subject of continuous research.

Over the past three decades, ECG delineation methods have focused on the proper design of pre-filters and comprehensive rules for the detection of peaks and isoelectric line crossings based on empirical thresholds for wave amplitudes, slopes, and time intervals [9,10,11,12,13,14,15,16,17,18,19]. The low computational resource requirements of threshold-crossing algorithms have made their implementation possible in ultra-low-power microcontrollers [16] and wearable devices with embedded FPGA boards [18].

The ECG delineation task has also been tackled by machine learning (ML) techniques. Some studies undertake a simple band-pass filter design for the purposes of ECG slope enhancement and the detection of representative samples belonging to P-waves, QRS-complexes, and T-waves by means of the K-nearest neighbor rule (KNN) [20,21,22] and support vector machines (SVMs) [22,23]. Others explore the potential power of more sophisticated preprocessing and delineation procedures, such as an extended Kalman smoother framework followed by a differential evolution algorithm [24]; a marginalized particle filter on a non-QRS signal and sequential Bayesian detection estimation algorithm [25]; a model based on Hermite and sigmoid functions combined with piecewise polynomial interpolation for the segmentation and low-dimensional representation of individual ECG beat segments [26]; a bidirectional hidden semi-Markov model based on the probability distributions of ECG waveform duration [27]; a multiscale morphological derivative transform-based technique [28], etc.

Given that periodic P-QRS-T patterns contain waves with different frequency content, their convolution with wavelet transform (WT) mother functions extracts time scale features with different resolutions that have been shown to be feasible for ECG delineation [6]. The first WT-based ECG delineator was presented by Li et al. in 1995 [29]. A common WT approach applies ECG decomposition and subsequent reconstruction from different decomposition levels to obtain sub-signals in frequency bands representing only QRS-complexes or P- and T-waves [30,31,32,33,34]. A similar effect was achieved by Yochim et al. [35], who applied a continuous WT with a varying scale of the mother wavelet for the subsequent delineation of QRS-, T-, and P-waves. Another approach applied WT reconstruction for R-wave enhancement and a subsequent search within an R-wave vicinity of the QRS boundaries, further P- and T-peaks, onsets, and offsets based on time and amplitude criteria in the ECG signal [36] or in higher WT scales [6,37,38,39]. In some studies, WT was applied as part of more complex ECG delineation procedures, e.g., De Lannoy et al. [40] used WT for strong ECG pre-filtering followed by a hidden Markov modeling approach for ECG delineation; Sehirli et al. [41] combined WT with moving average and zero-phase filters for ECG delineation and a KNN rule for the detection of Q-, R-, and S-waves; Fu et al. [42] applied a feature, referred to as a randomly selected wavelet transform feature and a random forest classifier adapted to infer the positions of the ECG characteristic points; Ghaffari et al. [43] used additional calculations over the ECG WT representation such as area–curve length to detect the R-peaks by variable thresholding and subsequently to identify P- and T-peaks and waves’ boundaries through amplitude and slope criteria.

In the past few years, there has been a noticeable increase in projects for ECG delineation that are related to the rapid development of deep neural network (DNN) technologies. Studies classified the input raw ECG samples as belonging either to P-wave, QRS-complex, T-wave, or no-wave by using different combinations of network layers. Common model architectures are composed of stacked non-linear convolutional filters with or without combination with temporal features extracted by recurrent layers that are either long short-term memory (LSTM) [44], bidirectional LSTM (BiLSTM) [44,45,46,47,48,49], or gated recurrent units (GRUs) [50]. Some networks keep an unchanged dimensionality of the raw ECG input in hidden and output layers [43,44,45,46], while others use bottleneck architectures, which downsample the input dimensionality by pooling operations and fully connected layers with a few output units [44,51,52]. This bottleneck approach compresses the input feature map and reduces the size of the trainable parameters in hidden layers but also extracts more abstract representations of the input raw ECG signals. High-level hierarchical features encode information important to the ECG signal itself, ignoring non-essential data, such as disturbing noises. This is demonstrated in denoising autoencoders that use symmetric architectures of encoding and decoding layers to reconstruct the input to a noise-free output under strong noisy conditions with variable frequency ranges and signal-to-noise ratios [53,54,55]. Recently, the effective functionality of encoder–decoder transformations has also been demonstrated for ECG signal segmentation. Most such studies found in the literature use U-net architectures, where the feature sets at each level of the encoder path are concatenated to the same level in the decoder path [56,57,58,59,60,61,62,63,64,65,66]. Thus, the size of the feature map in the decoder path is doubled with the idea to perceive both low- and high-level hierarchical features in the network, mitigating the potential information loss caused by the pooling operation in the encoder path. Additionally, some U-net architectures are designed to add input to output feature maps by using residual (skip) connection blocks in encoder and decoder paths [62,64], given that this technique has been shown to facilitate the training of DNNs and could solve a potential problem of vanishing gradients [67]. There are also different modifications that extend the basic U-net architecture, such as the doubled U-net structure in W-net [66] and the use of multi-heard self-attention modules [63,66]. The real need for various additional connections between hidden layers in the encoder–decoder transform NN for ECG delineation has not been thoroughly investigated in the literature so far.

With respect to the input receptive field, studies on ECG delineation handle different sets of input ECG leads, most of which use only one lead [6,13,14,15,16,17,18,20,24,25,29,30,31,32,33,36,39,40,41,42,43,44,45,46,48,49,50,51,52,58,59,60,61,62,63,66] or a combination of different lead pairs [47,59,64,65] mainly founded by the largest publicly accessible ECG databases with precise delineation labels in the PhysioNet database. Fewer studies have been found to use the three vectorcardiogram leads [9,37,57] and the standard 12-lead ECG [9,10,19,21,22,23,35,37,38]. The majority of cited studies process long-term raw ECG input (e.g., 10 s), with the exception of [14,19,26,34,44,52,59,65], where short beat segments or average heartbeats are subjected to delineation. The latter approach supports the robust measurement of P-, QRS-, and T-onsets, offsets, and peaks that could be further involved in methods for the detection of myocardial infarction [19], reduced ejection fraction [68], arrhythmia classification [14,69], etc.

Recently, Bock et al. [70] reported considerable inter-investigator variability in the assessment of ECG time domain parameters (P-, QRS-, and T-durations, and PQ- and QT-intervals), e.g., with a Q span ranging from 39 to 99 ms. That might be considered as a factor to compromise ECG-associated diagnosis. The objectivity of computer algorithms is underlined as their main advantage, given that the ECG parameters are determined on a “blinded” basis. However, the algorithms are limited to detect ECG patterns that they have been programmed for. Therefore, the extension of the training datasets is decisive for obtaining a more generalized performance, and this need is even more binding in ECG delineators based on DNNs.

The aim of this study is to develop a DNN-based framework for 12-lead ECG delineation that can fairly compare different encoder–decoder architectures applied on clinical and standard ECG databases and noise tests. We introduce an advanced approach for the machine-based supervised learning of DNNs with large ECG databases with extensive arrhythmia records, in contrast to traditional human-based supervised learning relying on limited annotated data. Thus, different DNN architectures can be compared in terms of their capability to reproduce the performance of a commercial ML algorithm for the measurement of the P-wave, QRS-, and T-wave boundaries in 12-lead ECG representative beats. These measurements are routinely used in clinical practice to support diagnostic decisions made by cardiologists, who can rely on the same commercial ECG system as the one used for supervised learning in this study. As a result of this study, we have concluded which are the best-performing DNN architectures, based on validation against the standard practices for the evaluation of diagnostic electrocardiographs in terms of the accuracy of interval measurements on biological ECGs (P-duration, PQ-interval, QRS-duration, and QT-interval). Additionally, robustness to noise and comparison against performances of other published studies are disclosed.

## 2. Materials and Methods

### 2.1. Representative Beat and Reference Measurements

The signal averaging of repetitive PQRST patterns is a widely used technique for noise reduction [71]. Thus, the conventional diagnostic practice based on resting ECG relies on interpreting a representative beat derived by the signal averaging of multiple cardiac cycles with similar characteristics from the entire recording, rather than interpreting single beats. This is because individual beats may be influenced by artifacts or may depend on momentary conditions (such as variations related to breathing) [4]. Essential to achieving accurate diagnostic conclusions is the precise computation of the representative beats on a beat-by-beat basis. First, the combined beats should exhibit similar morphology, characterized by the highly correlated waveforms of PQRST patterns [72]. Second, it is crucial to maintain the time alignment of individual beats to accurately reproduce the amplitudes of the PQRST waveform patterns. Common practices for time alignment include methods such as maximizing correlation, identifying maximal peaks, minimizing amplitude differences, and utilizing least squares techniques [73].

**Figure 1:**
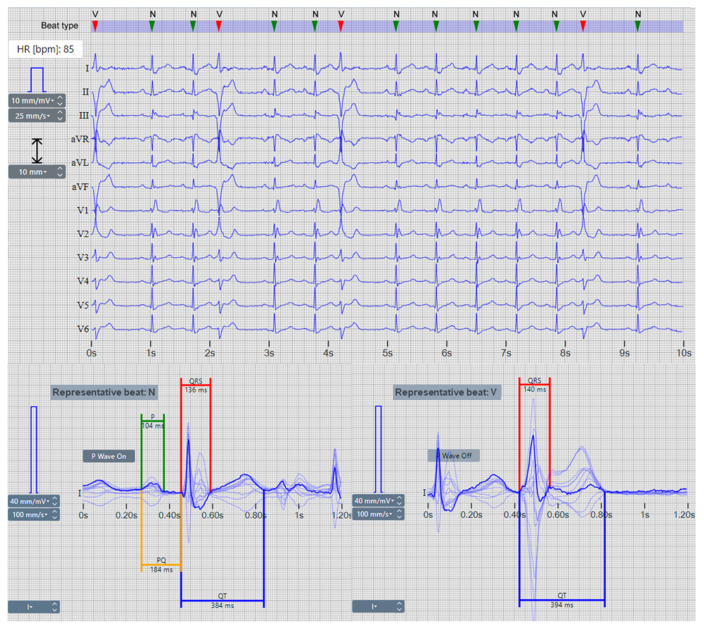
An illustration of the reference annotation process managed by the ECG Treatment Module (ETM, Schiller AG, Switzerland). **On top**: A 10 s rhythm of a 12-lead resting ECG record (HR00255 in PTB-XL database with original diagnosis “Incomplete bundle branch block”) and beat markers identifying two types of detected beats—N (normal sinus beat) and V (premature ventricular beat). **On the bottom**: The 12-lead ECG representative beats (duration of 1.2 s) computed for the detected two beat types—N (**left**) and V (**right**). The reference annotations for the presence of a P-wave (P-wave On/Off), fiducial points and durations of the P-wave (P, green), PQ-interval (PQ, yellow), QRS-complex (QRS, red), and QT-interval (QT, blue) are measured by the ETM.

In this study, the computation of the representative beats was facilitated by means of an existing ECG processing library by SCHILLER (ETM-2.6.5, Schiller AG, Baar, Switzerland) [74]. Utilizing multi-lead ECG rhythm input, the ETM performs heartbeat classification and generates ECG beats corresponding to each detected heartbeat morphology in the record. The representative beats are extracted from the input lead set within a 1.2 s window, sampled at 500 Hz, while ensuring consistent time alignment across all ECG leads. An example of ETM-based representative beats for two heartbeat classes (normal and ventricular beats) detected in a 10 s rhythm of resting 12-lead ECG is illustrated in Figure 1. When the ECG exhibits short RR-intervals, there is a possibility that the representative beat window may encompass segments of adjacent heartbeats, as illustrated for the premature ventricular beat in Figure 1 (right plot).

The ETM-based delineation of the representative beat provides fiducial points of the PQRST pattern, including P-onset, P-offset, QRS-onset, QRS-offset, and T-offset. These are named global representative beat fiducial points, which are common to all ECG leads. They are used for the calculation of global representative beat measurements covered by the standard IEC 60601-2-25:2011 [8], including four diagnostic wave durations and intervals: P-wave (P), PQ-interval (PQ), QRS-complex (QRS), and QT-interval (QT), as depicted by line segments in Figure 1. The Schiller ETM library is validated in Kligfield et al. [75].

### 2.2. Training Setting

#### 2.2.1. Training Database

The learning phase of this study uses the PhysioNet PTB-XL ECG database from the Physikalisch-Technische Bundesanstalt, version 1.0.1 [76,77], which is one of the largest freely accessible clinical 12-lead ECG waveform datasets, comprising 21,837 records from 18,885 patients. The twelve standard ECG leads (I, II, III, aVL, aVR, aVF, V1–V6) were recorded at rest for 10 s into a binary format with 16-bit precision at a resolution of 1 μV/LSB and sampling frequency of 500 Hz. The database was released with the primary purpose for the evaluation of machine learning algorithms, making certain that the whole database is a rich representation of healthy controls and pathologic ECG rhythms. This study considers all records without stratification to the diagnostic label. Nevertheless, it is worth noting that the reliability of the training results was justified with the variety of clinical rhythms available, including annotations for sinus rhythm (16,782 recordings), sinus bradycardia (637), atrial fibrillation (1514), atrial flutter (73), sinus tachycardia (826), sinus arrhythmia (772), supraventricular arrhythmia (157), supraventricular tachycardia (27), paroxysmal supraventricular tachycardia (24), normal functioning artificial pacemaker (296), bigeminal (82) and trigeminal pattern (20), ventricular premature complex PVC (1146), premature complex (10), and atrial premature complex PAC (398), where each recording could have one or several diagnoses [77].

The ETM was used for the extraction of representative beats and reference measurements from the PTB-XL ECG database, according to Section 2.1. Multiple representative beats may be extracted from one recording to represent all beat types detected by the ETM within the original 10 s rhythm, as demonstrated in the example in Figure 1. This includes both normal and ventricular ectopic beats observed in a single recording. A total of 24,232 representative beats were extracted, from which 20,955 beats (86.5%) were identified with a P-wave, and 3277 beats (13.5%) were identified without a P-wave (such as atrial fibrillation beats, ventricular beats, paced beats, nodal beats, etc.).

#### 2.2.2. Training Phase I: Delineation Model

ECG delineation is performed using a DNN model, which takes as input raw data samples from a 12-lead representative beat and produces probabilities for each input sample, indicating its association with one of three fundamental ECG waves/intervals: P-wave, QRS-complex, and QT-interval.

The input data are configured as a tensor with size (W × Ch_in), where

- W = 512 represents the analysis window (samples), including a representative beat with a duration of 1.024 s (sampling frequency of 500 Hz).
- Ch_in = 13 represents the number of input channels, including 12 channels for standard 12 ECG leads (I, II, III, aVR. aVL, aVF, V1–V6) and 1 channel for a linear time vector (t = 1, 2, …, 512).

Our measurement setting uses an analysis window with a central representative beat and related global annotations. However, waves of preceding and/or following beats with RR-intervals shorter than 1 s may also appear in the analysis window. Such a phenomenon is common in rapid heart rates or premature ectopic beats (see the representative beat V in Figure 1, bottom). By consent, side heartbeats are not a subject of measurement and annotation in the representative heartbeat analysis. Therefore, the time vector channel contains information that can be useful to guide the training of the DNN model on the location of the waves that need delineation. To prevent overtraining on a specific QRS-detector and representative beat synchronization, we utilize a random time offset generator. This tool reads representative beats with varying time shifts up to ±100 ms.

**Figure 2:**
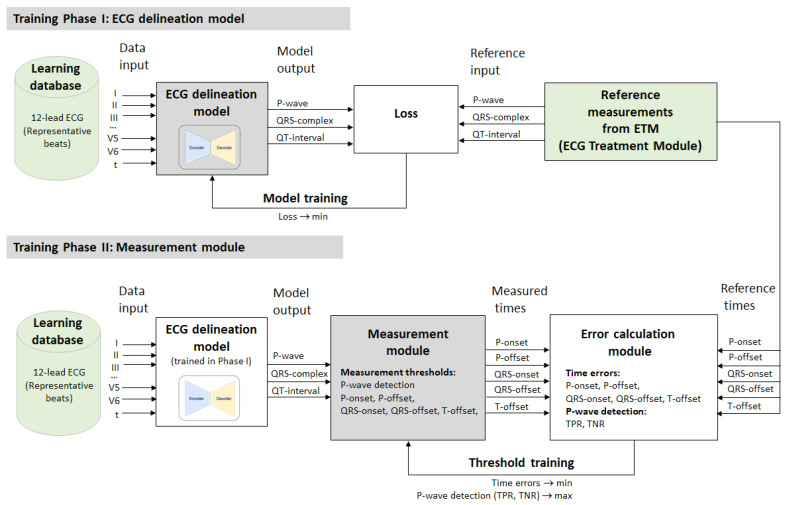
The training concept for the delineation of representative beats in 12-lead ECG, including the following: the Phase I training of the ECG delineation model (**top**) and Phase II training of the threshold-based Measurement module (**bottom**). ETM: ECG Treatment Module (Schiller AG, Switzerland); TPR: true positive rate; TNR: true negative rate.

The training of the DNN model for ECG delineation, also named Phase I training, is depicted in Figure 2 (top). It is supervised by the reference measurements from the ETM diagnostic module in the learning PTB-XL dataset. The ETM provides the global representative beat fiducial points of P-onset, P-offset, QRS-onset, QRS-offset, and T-offset, but these are not directly used as the reference input to the model trained for regression. Instead, we found that the most effective training is achieved by solving a binary segmentation problem for each of the following three segments: P-wave, QRS-complex, and QT-interval. As a result, the output of our DNNs is a tensor of size (W × 3). As a loss function for the training, we use the binary cross-entropy loss:

(1) $$Loss=-\frac{1}{W}\sum_{i=1}^{W}(x_{i}\cdot log({\hat{x}}_{i})+\left(1-x_{i}\right)\cdot log(1-{\hat{x}}_{i})),$$

where W is the number of input samples per representative beat, $x_{i}$ is the reference binary input, and ${\hat{x}}_{i}$ is the predicted output.

**Figure 3:**
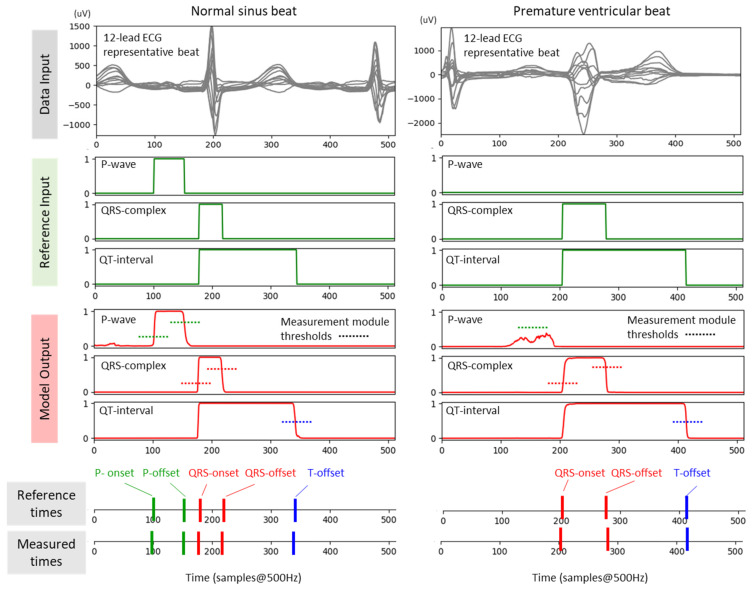
An illustration of the data used in the training phase in Figure 2, including two representative beats: normal sinus beat (recording HR12720 in PTB-XL database) and premature ventricular beat (recording HR11445 in PTB-XL database). The time scale (x-axis) in all plots corresponds to the size of the data frames as defined by the ECG sampling frequency of 500 Hz and representative beat duration of 512 samples (1.024 s). Data input (12 gray traces, overlapped): the representative beat seen in 12 standard ECG leads (I, II, III, aVR, aVL, aVF, V1-V6). Reference input (3 green traces): P-wave, QRS-complex, and QT-interval binary signals generated by the ETM (ECG Treatment Module) and used for model training in ECG delineation. Model output (3 red traces): the probabilistic response [0;1] for the presence of the P-wave, QRS-complex, QT-interval. Reference times (vertical lines): instants of P-onset, P-offset, QRS-onset, QRS-offset, and T-offset, determined at respective transition moments of the three reference inputs. Measured times (vertical lines): instants of P-onset, P-offset, QRS-onset, QRS-offset, and T-offset, determined at moments when the three outputs of the ECG delineation model cross the respective Measurement module thresholds.

The three target signals for the training are illustrated in Figure 3 (green traces), representing binary series [0, 1] with ones present throughout the duration of the segments of interest. The DNN outputs a probabilistic response, as shown in Figure 3 (red traces).

#### 2.2.3. Training Phase II: Measurement Module

In Phase II of the training, we address the challenge of interpreting the probabilistic outputs of the ECG delineation model. As shown in Figure 3, these outputs exhibit limited steepness on the rising and falling edges, leading to uncertainty in identifying the fiducial points of the waves’ onsets and offsets. According to the training scheme in Figure 2 (bottom), we use a Measurement module as a postprocessing step to the DNN to precisely measure the times of the P-onset, P-offset, QRS-onset, QRS-offset, and T-offset. They are identified by a threshold-crossing method that uses five thresholds, according to the illustration in Figure 3 (left): two thresholds for the rising and falling edges of the P-wave output (P-onset, P-offset); two related thresholds for the QRS-complex output (QRS-onset, QRS-offset); and one threshold for the falling edge of the QT-interval output (T-offset).

In Phase II, each threshold is trained to ensure that the fiducial point it measures at a given time ($T_{meas}$) closely matches the reference time ($T_{ref}$) provided by the ETM diagnostic module. The degree of alignment is assessed by the error calculation module depicted in Figure 2, which computes the time error (TE) in a representative beat (n)

(2) $$TE(n)=T_{meas}(n)-T_{ref}(n),$$

and further estimates the mean and the standard deviation of the time error for a number of N representative beats in the training database:

(3) $$mean(TE)=\frac{\sum_{n=1}^{N}TE\left(n\right)}{N},std(TE)=\sqrt[{}]{\frac{\sum_{n=1}^{N}{\left(TE(n)-mean(TE)\right)}^{2}}{N-1}}$$

The Phase II training process independently adjusts each of the five thresholds, ensuring a zero mean time error (mean(TE)→0) for P-onset, P-offset, QRS-onset, QRS-offset, and T-offset, respectively. Although a low TE standard deviation is also a goal $\left(std\left(TE\right)\to min\right)$, it cannot be essentially controlled, but rather, $std\left(TE\right)$ is reported as a measure of the variance in or reliability of the measurement process. In order to eliminate the influence of outliers or data points on the tails that may unfairly affect the arithmetic mean, Equation (3) is estimated for N beats representative to the 95th percentile of TE in the training dataset. This approach is in line with the recommendations for reporting steady working automatic algorithms for QT-interval measurement in [78].

An additional P-wave detection threshold is defined, considering a positive P-wave detection in case the DNN output equals or exceeds the threshold and a P-wave rejection in case the DNN output does not reach the threshold. The threshold is adjusted by the Receiver Operating Characteristic (ROC) curve on the total training dataset to provide maximal balanced performance between the true positive rate (TPR) and the true negative rate (TNR):

(4) $$TPR=\frac{TP}{TP+FN},TNR=\frac{TN}{TN+FP},TPR+TNR\to max,$$

where TP (true positives) and FN (false negatives) are the detected and rejected P-waves, respectively, in representative beats with P-waves; TN (true negatives) and FP (false positives) are the rejected and detected P-waves, respectively, in representative beats without P-waves. Note that the evaluation of P-wave detection performance relies on the TPR and TNR. To ensure that P-wave onset/offset measurements are not biased by wrong P-wave detections, TE (P-onset) and TE (P-offset) are calculated using Equation (3) exclusively for true positive P-waves.

### 2.3. Test Setting

#### 2.3.1. Test Database Used with Reference ETM Measurements

We used a public test database from the PhysioNet database, specifically the PTB diagnostic ECG database. Since the initial release of 549 resting 15-lead ECG recordings (standard 12-leads and 3 Frank leads) from 290 subjects by the Physikalisch-Technische Bundesanstalt in 2004 [79,80], this database has been widely acknowledged in ECG signal processing research and algorithmic benchmarking. It stands out for the unique representation of healthy controls and a variety of pathologies, including myocardial infarctions, arrhythmias, heart blocks, myocardial hypertrophy, etc. Notably, this database does not overlap or share records with the training PTB-XL dataset [77]. 

The test phase of this study used a subset of 516 ECG records (male: 377, female: 139) from the version of the PTB diagnostic ECG database included in the Physionet/Computing in Cardiology Challenge 2020 [81]. The ETM was used for the extraction of 12-lead ECG representative beats and reference measurements according to Section 2.1. More than one representative beat might be extracted from one recording, given that they are characteristic to all beat types detected by the ETM in the original 10 s rhythm. A total of 603 representative beats were extracted, from which 527 beats (87.4%) were identified with a P-wave, and 76 beats (12.6%) were identified without a P-wave (such as atrial fibrillation beats, ventricular beats, paced beats, nodal beats, etc.). They were used for the computation of the P-wave detection performance by Equation (4).

The fidelity of measurements for P-onset, P-offset, QRS-onset, QRS-offset, and T-offset is checked against ETM annotations in 603 representative beats from the test set, applying Equations (2) and (3). A non-outlier range of mean ± three times the standard deviation is considered, assuming that the vast majority of cases (approximately 99.7%) fall within it in a normally distributed dataset [82]. Any cases lying outside this range are deemed extreme outliers, representing data points significantly divergent from the rest of the dataset, potentially skewing the analysis or affecting the validity of statistical measures. To mitigate their impact, outliers are capped to the non-outlier range, ensuring that their extreme values do not disproportionately influence the analysis. Consequently, we provide statistical measurements for all data points in the test database but do not apply more aggressive outlier removal techniques that result in data loss by dropping part of the observations.

#### 2.3.2. The Test Database Used According to the Standard-Based Evaluation Practice

Recommendations for accuracy reports of automatic ECG delineation algorithms specify maximum tolerances for acceptable measurement errors of four diagnostic wave durations and intervals, as illustrated in Figure 1 (P-duration, PQ-interval, QRS-duration, QT-interval). The corresponding duration errors (DEs) are computed as the difference between the automatic duration measurement ($D_{meas}$) and the reference duration value ($D_{ref}$), taken from the annotations of a particular record (n) in the test database:

(5) $$DE\left(n\right)=D_{meas}\left(n\right)-D_{ref}\left(n\right)$$

The accuracy metrics report the duration error mean value $\left(mean\left(DE\right)\right)$ and standard deviation $\left(std\left(DE\right)\right)$, computed for a set of N records in the test database, using the same Equation (3) as for TE.

According to the IEC 60601-2-25:2011 standard [8], the duration errors shall be evaluated for a set of N = 100 ECGs with the following sequential numbering in the Common Standards for Electrocardiography (CSE) database [83,84], including both physiological and pathological records with diverse ECG abnormalities: “MO1_” series {001–005, 007–009, 011–017, 019, 021, 022, 024–044, 046–049, 051, 053, 055, 058–066, 068, 069, 071–088, 090, 091, 095–099, 101–108, 110, 112–116, 118, 123–125}. In order to reject the influence of outliers in the record set n = {1, …, 100}, the eight largest deviations from the mean $\left|DE\left(n\right)-mean\left(DE\right)\right|$ shall be excluded, and the mean and standard deviation of the remaining differences shall not exceed the specified tolerances: P-wave DE (mean ≤ ±10 ms, std ≤ ±15 ms), PQ-interval DE (mean ≤ ±10 ms, std ≤ ±10 ms), QRS-complex DE (mean ≤ ±10 ms, std ≤ ±10 ms), QT-interval DE (mean ≤ ±25 ms, std ≤ ±30 ms). 

A useful practice covered in the former standard IEC60601-2-51:2003 [85] concerned a set of tests for the noise immunity of automatic ECG delineation algorithms. It was required that the stability of the interval and wave duration measurements must be tested with N = 10 selected ECGs in the CSE database (“MO1_” series {008, 011, 013, 014, 015, 021, 026, 027, 042, 061}) without noise and in the presence of three kinds of noises:

- A 25 μV r.m.s. high-frequency (HF) noise;
- A 50 μV peak-to-peak 50/60 Hz sinusoidal power-line (PL) frequency noise;
- A 1 mV peak-to-peak 0.3 Hz sinusoidal low-frequency (LF) baseline noise.

According to the standard [85], the duration error in the presence of noise (DE_noise_) is determined as the differences in duration measurements between noise-free ECGs and ECGs with each type of noise:

(6) $${DE}_{noise}\left(n\right)=D_{noise-free}\left(n\right)-D_{noise}\left(n\right)$$

Similar to the practice with noise-free records, the mean value and standard deviation of ${DE}_{noise}$ shall be calculated by Equation (3) after rejecting the influence of outliers, which are the two largest deviations from the mean. Only the results are reported, with no requirements set for acceptable limits. 

The described standard-based evaluation practice was applied in the test phase of the deep learning algorithms in this study. One representative beat per recording was taken, based on ETM calculations for the predominant beat class. The original annotations provided by the CSE database were taken as a reference.

### 2.4. ECG Delineation Model 

#### 2.4.1. Convolutional Encoder–Decoder Network (CED-Net)

Encoder–decoder architectures are often used in image and signal segmentation tasks [59,60,61,62,63,66,86]. They consist of an encoder part that downsamples the input data to extract high-level encoded features and a decoder part that upsamples these features to generate a segmentation mask. An important design consideration for the encoder–decoder is the model symmetry in terms of the total size of the feature maps and the number of neurons in all hidden layers in both the encoder and decoder parts. Another issue is the bottleneck encoding size, which must be sufficient to represent the input feature map from an informational point of view.

**Figure 4:**
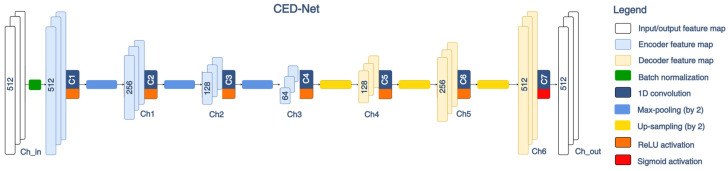
The architecture of the designed Convolutional Encoder–Decoder network (CED-Net).

This study uses an ECG delineation model based on Convolutional Encoder–Decoder Network (CED-Net) in Figure 4. As defined in Section 2.2.2, the input feature map is a two-dimensional tensor with size (W × Ch_in = 512 × 13), where W is the time axis dimension of the ECG representative beat, and Ch_in is the number of input channels, including 12 ECG leads and one time vector (t = 1, 2, …, 512). The Z-score normalization of the input data is provided with the first hidden Batch normalization layer. 

The CED-Net design in Figure 4 uses an encoder part with three convolutional blocks, which downsample the input feature map progressively from a size of 512 (input) to 256, 128, and 64 (bottleneck). One encoder convolutional block includes a 1D convolutional layer (C1, C2, or C3) with rectified linear (ReLU) activation, followed by a max pooling operation (pool_size = 2). The transformation of the encoder feature map $x_{i}$ after the i^th^ convolutional block can be represented as follows:

(7) $$x_{i}=maxpooling\left(ReLU\left(W_{i}\cdot x_{i-1}+b_{i}\right),pool\_size\right),$$

where (.) denotes 1D convolution operation; $\left(W_{i},b_{i}\right)$ is the matrix with weights and biases of the respective encoder convolutional layer; ReLU=max⁡(x,0) is the ReLU non-linear activation; maxpooling(x,pool_size) denotes a pooling operation that calculates the maximum value for “pool_size” temporal steps along the time axis of the feature map x.

The CED-Net decoder part mirrors the encoder by using three decoder convolutional blocks, which gradually increase the resolution of the feature maps from 64 (bottleneck) to 128, 256, and 512. One decoder convolutional block includes a 1D convolutional layer (C4, C5, or C6) with ReLU activation, followed by an upsampling operation (size = 2). The transformation of the decoder feature map $\hat{x_{i}}$ after the i^th^ convolutional block is denoted as follows:

(8) $$\hat{x_{i}}=upsampling\left(ReLU\left(\hat{W_{i}}\cdot \hat{x_{i-1}}+\hat{b_{i}}\right),size\right),$$

where $\left(\hat{W_{i}},\hat{b_{i}}\right)$ is the matrix with weights and biases of the respective decoder convolutional layer; $upsampling\left(x,size\right)$ denotes an operation that repeats each value of the feature map $\hat{x_{i-1}}$ “size” times.

The last convolutional layer (C7) transforms the CED-Net output according to the segmentation task defined in Section 2.2.2 and presented in Figure 3. The output feature map is provided in a tensor with size (W × Ch_out = 512 × 3), where W represents the same time axis dimension as the input ECG representative beat; Ch_out corresponds to the number of 3 output channels (P-wave, QRS-complex, QT-interval). The convolution C7 is followed by a sigmoid activation function to force the output z in the probability range *p* ∈ [0;1]:

(9) sigmoidz=11+e−z.

CED-Net trainable parameters are equal to the total number of weights and biases of all convolutional layers, which are determined by the corresponding kernel size (K) and number of channels (Ch):

(10) $$Trainable\;parameters=\sum_{i=1}^{N}{Ch}_{i}{(K}_{i}{Ch}_{i-1}+1),$$

where N = 7 convolutional layers (C1–C7); ${Ch}_{i=0}$ refers to the number of input channels.

The kernel size and number of hidden channels are the subject of model optimization, as further disclosed in the Section 3.

#### 2.4.2. Modifications of CED-Net Architecture with Residual and Recurrent Connections

**Figure 5:**
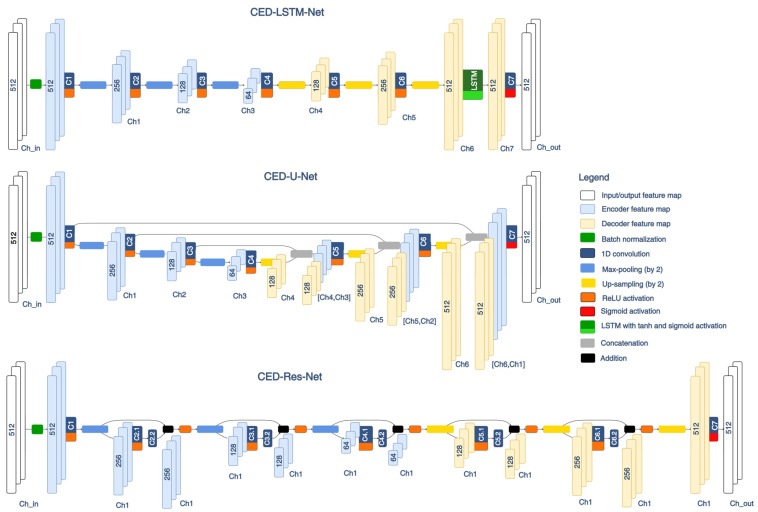
Modifications of the basic CED-Net architecture in Figure 4 by adding an LSTM layer (CED-LSTM-Net), U-net residual connections (CED-U-Net), and residual blocks (CED-Res-Net).

To explore the possibility of improving the performance of the basic CED-Net architecture, we propose three modifications by adding new layers or skip connections in the encoder–decoder path, presented in Figure 5. The structure of the new CED-Net architectures is motivated by recognized techniques that can either follow long-term dependencies (LSTM layers) or improve training by combining low- and high-level hierarchical features through residual connections (U-net and Res-net). Short descriptions of the networks are further presented.

1. CED-LSTM-Net (Figure 5, top): This is a CED-Net architecture with an LSTM layer provided in the decoder feature map to learn long-term dependencies in PQRST patterns. LSTM is a recurrent neural network layer with embedded memory cells, which act as accumulators of the state information [44]. The memory cells’ decision to forget or remember a certain part of the information in the input time series is regularized by input, forget, and output gates, activated by non-linear sigmoid and hyperbolic tangent functions. Our application uses one memory cell per channel, defined by the dimension of Ch6 in Figure 5.
2. CED-U-Net (Figure 5, middle): This is a CED-Net architecture with residual connections between symmetrical encoder and decoder feature maps, typical for U-nets [87]. Thus, the convolution is applied on concatenated information from low- and high-order feature maps:
  (11) $$D_{i+1}=ReLU\left(W\cdot \left[D_{i},E_{j}\right]+b\right),$$
  where $\left[D_{i},E_{j}\right]$ means the concatenation of symmetrical decoder and encoder feature maps, denoted as [Ch4,Ch3], [Ch5,Ch2], and [Ch6,Ch1] in Figure 5.
3. CED-Res-Net (Figure 5, bottom): This is a CED-Net architecture, including a sequence of five residual blocks. In the literature, a residual block takes an input x and produces an output F(x) + x by elementwise addition, followed by a non-linear activation function, usually ReLU [67]. In our design, F(x) represents a sequence of two convolutions, and the output y can be presented as follows:
  (12) $$y=ReLU\left(W_{2}\cdot ReLU\left(W_{1}\cdot x+b_{1}\right)+b_{2}+x\right),$$
  where $\left(W_{1},b_{1}\right),(W_{2},b_{2})$ are the matrices with weights and biases of the first and second convolution of the residual block, respectively.

## 3. Results

**Figure 6:**
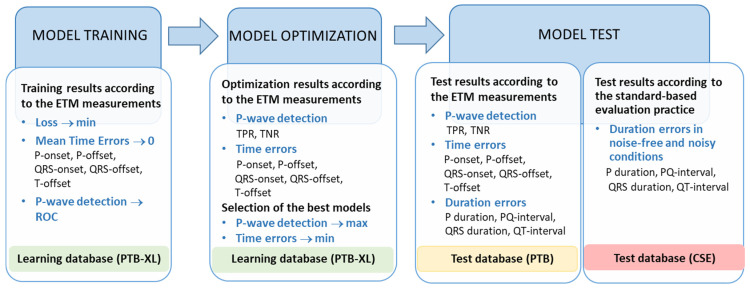
An illustration of the results’ flow associated with the training, optimization, and testing of the developed ECG delineation models.

This section presents different results obtained in the training, optimization, and test phases of the developed CED-Net architectures in the ECG delineation task. To facilitate the understanding of such a relatively large amount of information, we illustrate the global flow of results in Figure 6. It is organized in three subsections:

1. Model training: Shows the training results for a single model in order to illustrate the global principle of training all models.
2. Model optimization: Derives trained models according to specific hyperparameter optimization; considers a relative ranking of trained models based on their performance in the training dataset; selects the best trained models.
3. Model test: Presents the results of the best trained models with two types of tests applied on independent test datasets:
  (1) Test results according to the ETM measurements;
  (2) Test results according to the standard-based evaluation practice.

### 3.1. Model Training

#### 3.1.1. Phase I Training

This section explains the learning strategy of a CED-Net model included in the Phase I training scheme for ECG delineation in Figure 2. The iterative training process evaluates the loss by Equation (1) in two randomly shuffled subsets of the training PTB-XL database: the training subset (70%) and validation subset (30%). These two subsets proportionally reflect the statistical characteristics of the training database, which is an essential requirement to have validation data representative of the training data. The training loss is used to estimate how well the model fits its hidden neural weights and biases to the training data, while the validation loss estimates how well the trained model fits new data. In general, the learning curves show the trends of training and validation losses over iterative training epochs and are used to determine the optimal model fit (minimal validation loss), given that scenarios for model underfit and overfit should be avoided. Underfitting occurs either when the model is unable to accurately represent the training data (both training and validation are high) or when the validation loss decreases but has not reached a minimum. Overfitting is observed when the model performs well on training data (training loss is decreasing) but poorly on the validation set (validation loss is increasing or stagnating). Therefore, our study follows the training strategy with early stopping if the validation loss does not improve for a preset epoch tolerance. Furthermore, dropout (rate = 0.1) is applied to each convolutional layer as a regularization tool to prevent overtraining on specific data by randomly setting a fraction of the neurons to zero during training.

**Figure 7:**
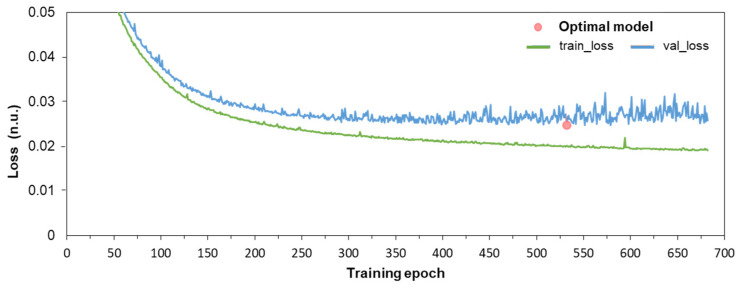
Learning curves of a CED-Net model for ECG delineation, presenting the change in the training loss (train_loss) and validation loss (valid_loss) over training epochs. The validation loss at epoch 532 is highlighted to represent the selected trained model with optimal fit (val_loss → min).

Figure 7 illustrates the learning curves of a CED-Net model whose training was initiated for a maximum of 1000 epochs with an early stopping tolerance of 150 epochs. Both training and validation losses are smoothly decreasing and show no findings for underfitting and overfitting. The training was stopped after 682 epochs, given that the minimum validation loss was found at epoch 532. This model was considered optimally trained and was used for further evaluation steps.

#### 3.1.2. Phase II Training

This section explains the strategy for the training of the Measurement module thresholds included in the Phase II training scheme for ECG delineation, depicted in Figure 2 and defined in Section 2.3.2. 

**Figure 8:**
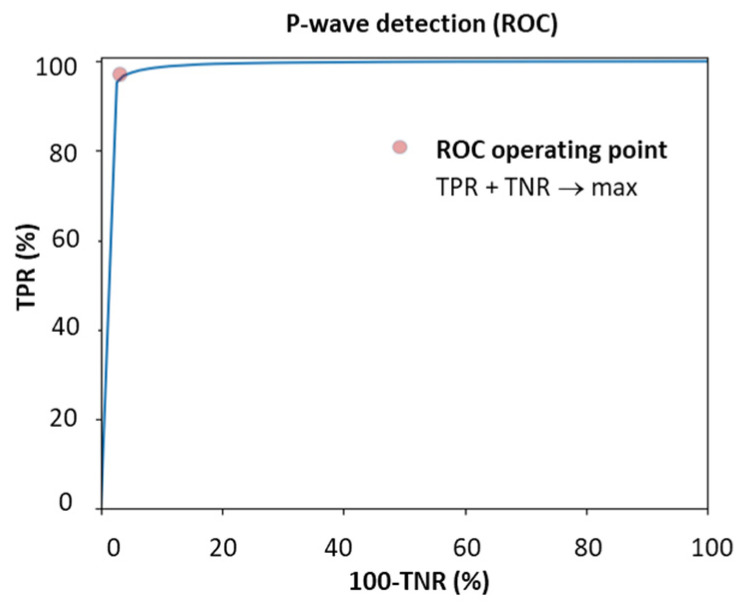
ROC curve for P-wave detection, illustrating the training of the measurement threshold applied to the CED-Net output (P-wave) to ensure TPR + TNR → max in the training database (PTB-XL). TPR: true positive rate, TNR: true negative rate.

The P-wave detection threshold is defined using the learning ROC curve in Figure 8. This ROC curve represents the relation between the TPR and TNR for P-wave detection, both calculated for different values of the threshold applied to the CED-Net output (P-wave). The operating point is selected for providing balanced performance: TPR + TNR → max.

**Figure 9:**
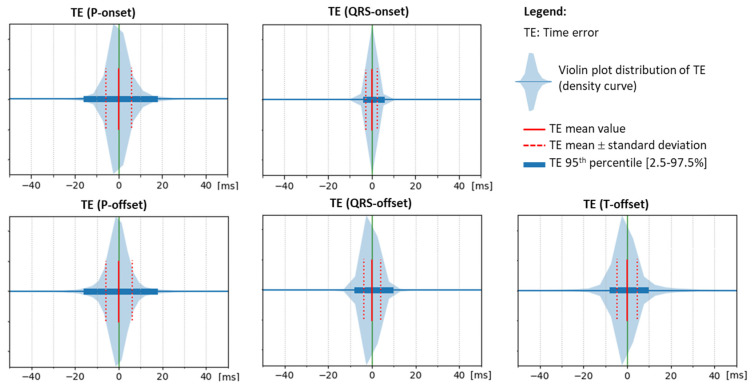
Violin plot density distributions of five time errors: TE (P-onset), TE (P-offset), TE (QRS-onset), TE (QRS-offset), and TE (T-offset), illustrating the training of the measurement thresholds applied to the CED-Net outputs (P-wave, QRS-complex, QT-interval) to ensure mean time errors → 0 in the training database (PTB-XL). The distributions are additionally indicated by the ranges of the mean ± standard deviation computed for the 95th percentile of TE.

The five thresholds for the detection of the P-onset, P-offset, QRS-onset, QRS-offset, and T-offset in the respective CED-Net outputs are adjusted to provide a zero mean time error (mean(TE)→0) in the training database. This is justified in Figure 9, which illustrates the density distributions of the corresponding time errors, showing a relatively symmetrical spread around 0. The width of TE distributions is quantified by the 95th percentile and standard deviation, considering that the lower they are, the higher the reliability of the measurement process. Deduced from the graphs, the training was the most reliable for the QRS-onset and the least reliable for the P-onset and the P-offset. 

### 3.2. Model Optimization

#### 3.2.1. The Number of Channels and Kernel Size of the Basic CED-Net Architecture

**Figure 10:**
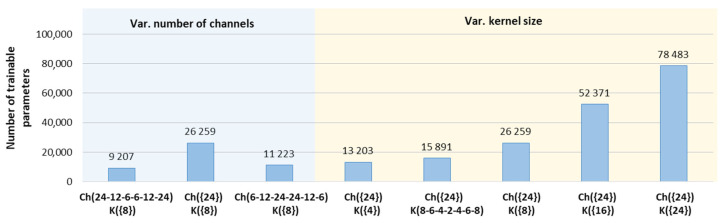
The number of trainable parameters of CED-Net models generated to optimize the hyperparameters of convolutional layers in the architecture of Figure 4. On the left (blue section): models with a variable number of channels (Ch1 to Ch6). On the right (yellow section): models with a variable kernel size (K) of seven 1D convolutions (C1 to C7). In the x-label, the values in brackets apply to all layers, specified as a series of numbers or as a single numeric constant for all layers.

The basic CED-Net architecture in Figure 4 is optimized by setting variable numbers of channels (Ch1–Ch6) and kernel sizes (K1–K7) for sequential convolutional layers (C1–C7), as indicated in the x-axis labels of Figure 10. 

The first-level CED-Net optimization uses a fixed kernel size K({8}) for all convolutions in Figure 4 and explores three strategies for setting variable channels:

- Ch({24}) denotes a fixed number of 24 channels for Ch1–Ch6, selected to double the number of ECG leads to prevent information loss;
- Ch(24-12-6-6-12-24) and Ch(6-12-24-24-12-6) respectively indicate a decreasing and increasing number of channels from bottom to top of the encoder and mirroring them in the decoder.

**Figure 11:**
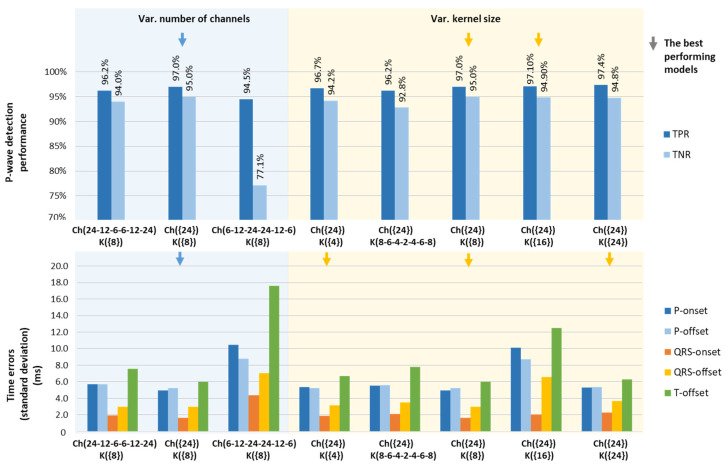
Training performance of CED-Net models subjected to hyperparameter optimization of convolutional layers, as explained Figure 10. **On top**: P-wave detection performance. **On bottom**: Time errors (standard deviations) of five fiducial points. Models with best training performances on top (TPR → max, TNR → max) and bottom (time errors → min) are marked with arrows.

The numbers of trainable parameters in Figure 10 (blue section) indicate that increasing and decreasing numbers of channels create models that are, respectively, from 2.3 to 2.9 times lighter than the redundant model with fixed 24 channels. Nevertheless, our optimization strategy selects the best-performing model Ch({24}), which is highlighted in Figure 11 (blue arrow), presenting maximal P-wave detection performance (TPR = 97%, TNR = 95%) and std$\left(TE\right)\to min$ (P-onset = 4.9 ms, P-offset = 5.3 ms, QRS-onset = 1.7 ms, QRS-offset = 3 ms, T-offset = 6 ms).

The second-level CED-Net optimization uses the highlighted above optimal number of channels Ch({24}) and explores various kernel sizes:

- K({4}), K({8}), K({16}), and K({24}) denote a fixed number of 4, 8, 16, and 24 kernels for the seven 1D convolutions (C1–C7) shown in Figure 4;
- K(8-6-4-2-4-6-8) indicates a decreasing number of kernels from the bottom to the top of the encoder and mirroring them in the decoder of Figure 4.

Derived from Equation (10), the number of trainable parameters in Figure 10 (yellow section) is linearly dependent on the kernel size. Therefore, a stepwise increase in kernel size (from 4 to 24) allows for the comparison of models with gradually increasing complexity. Although the complexity range of the models is up to six times (from 13,203 to 78,483 parameters), we have not observed substantial differences in their training performance, presented in Figure 11 (yellow section). Nevertheless, the best P-wave detection, $\left(TPR,TNR\right)\to max$ is highlighted for two models K({8}), K({16}). The minimum time error, std$\left(TE\right)\to min$ is marked for three models K({4}), K({8}), and K({24}). Thus, the K({8}), Ch({24}) model performed the best with both optimization criteria, being balanced in terms of training parameters, hence our final choice for the optimal CED-Net hyperparameters.

#### 3.2.2. Learning Rates of Different CED-Net Architectures

**Figure 12:**
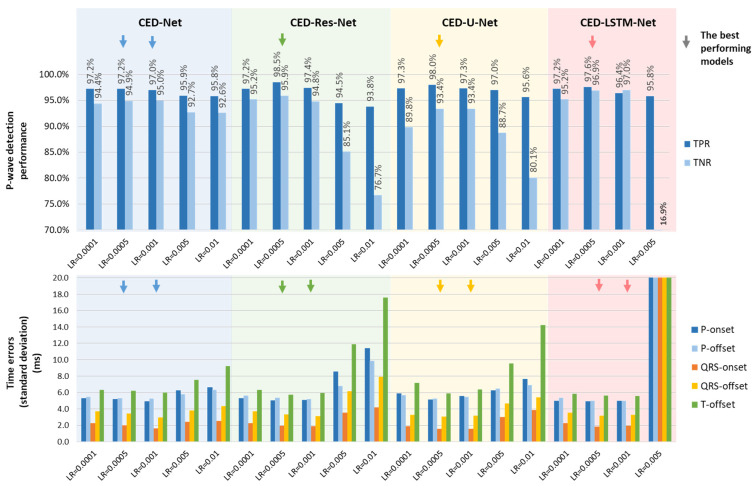
Training performance of CED-Net, CED-Res-Net, CED-U-Net, and CED-LSTM-Net architectures for five learning rates LR = {0.0001, 0.005, 0.001, 0.005, 0.01}. **On top**: P-wave detection performance. **On bottom**: Time errors (standard deviations) of five fiducial points. Models with best training performances on top (TPR → max, TNR → max) and bottom (time errors → min) are marked with arrows.

The CED-Net optimal hyperparameters K({8}), Ch({24}) are applied to the architectures in Figure 5, resulting in four models ordered by complexity (number of trainable parameters): CED-Net (26,259), CED-LSTM-Net (30,963), CED-U-Net (36,051), and CED-Res-Net (49,419). Given the different layers and connectivity, it can be assumed that models can be optimally trained with specific learning rates. Therefore, five learning rates LR = {0.0001, 0.005, 0.001, 0.005, 0.01} are used for the comparative study in Figure 12. Note that CED-LSTM-Net (LR = 0.01) is not shown because it failed to train in several independent runs. The best trained models are highlighted for each architecture, noting that LR = 0.005 and LR = 0.001 are almost equally effective for training all models in terms of std$\left(TE\right)\to min$, while LR = 0.005 provides the best P-wave detection $\left(TPR,TNR\right)\to max$. Therefore, our final choice for the best model representative from each architecture is trained with LR = 0.005.

### 3.3. The Test Results According to the Reference ETM Measurements

Applying the test concept in Section 2.3.1 and the test scheme in Figure 6, we reported an independent test evaluation of the best trained models (CED-Net, CED-Res-Net, CED-U-Net, and CED-LSTM-Net) in Table 1. All models present P-wave detection performance in the narrow range (94.7–97.9%), as well as very similar ranges for the time and duration errors with respect to mean values (−2.6 to 2.4 ms) and standard deviations (2.9 to 11.4 ms).

### 3.4. The Test Results According to the Standard-Based Evaluation Practice

Using the test concept outlined in Section 2.3.2 and the test scheme illustrated in Figure 6, we have evaluated the performance of the best trained models (CED-Net, CED-Res-Net, CED-U-Net, and CED-LSTM-Net) in delineating diagnostic wave durations and intervals (P-duration, PQ-interval, QRS-duration, QT-interval) in accordance with the standard-based guidelines using the CSE dataset. The results are presented in Table 2 and can be directly compared with the standard tolerances for the mean value and standard deviation [8]. Although most of the measurements are within the tolerances, we have found that the CED-Res-Net, CED-U-Net, and CED-LSTM-Net models are not compliant with the standard (±10 ms), presenting up to 4 ms larger standard deviations for PQ-interval measurements. As an alternative, the CED-Net model meets all standard tolerances.

**Figure 13:**
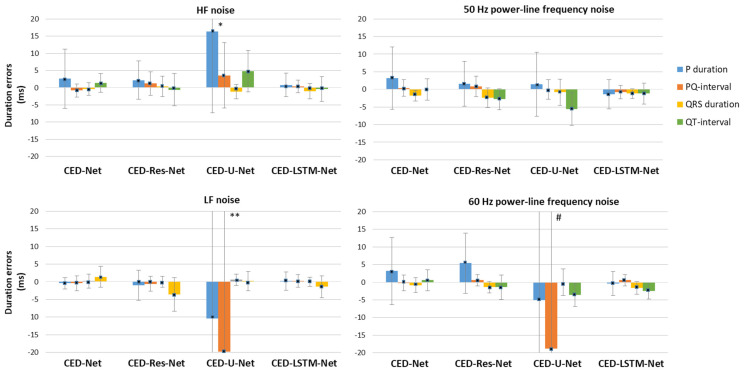
Duration errors in presence of noise estimated for test CSE dataset. Data show differences in wave durations in absence of noise vs. four types of added noises, depicted as mean values (color bars + dots), and standard deviations (whiskers). Extremity duration errors, appearing outside the defined y-range (±20 ms), are observed for CED-U-Net architecture as follows: * HF noise (−16.4 ± 23.7 ms for P-duration), ** LF noise (−10.4 ± 37.8 ms for P-duration, −19.8 ± 64.7 ms for PQ-interval), and # 60 Hz power-line frequency noise (−5 ± 42.7 ms for P-duration, −18.8 ± 65.2 ms for PQ-interval).

Figure 13 compares the four network models in terms of their robustness to four types of noise: HF, LF, and PL noise at 50 Hz or 60 Hz, added to representative ECGs from the CSE database, as defined in Section 2.3.2. It is worth noting that these results can be interpreted for relative comparisons between models as no standard requirements have been set for this test in noisy conditions. According to Equation (6), the duration measured in the presence of noise is subtracted from that in the absence of noise, yielding a signed duration error. The sign indicates the effect of the noise: a positive value implies a shortened duration measurement, while a negative value implies a prolonged duration. Nevertheless, an error close to zero indicates a robust model with stable duration measurements unaffected by the presence of specific noise type. The data in Figure 13 give evidence that three of the models (CED-Net, CED-Res-Net, and CED-LSTM-Net) are robust to all types of noise, presenting a mean duration error below ±2.5 ms that might reach up to +5 ms (prolonged P-duration) for HF and PL noises. The standard deviations of the errors are under 8 ms. In contrast to other models, CED-U-Net presents extremity errors (means up to 20 ms, standard deviations up to 65 ms), associated with the following: P-wave prolongation/shortening for HF/LF noise and QRS shortening (LF and PL 60 Hz noise). Such reduced noise immunity can be attributed to the specific U-net skip connections, which directly transfer information from input to output without passing convolutional filters.

### 3.5. Computing Resources

Deep learning on a PC requires powerful computing resources, so the experiments were conducted on a GPU-based workstation PERSY Stinger with Intel CPU Xeon Silver 4214R @ 2.4 GHz (2 processors), 96 GB RAM (Intel, Santa Clara, CA, USA), and NVIDIA RTX A5000-24 GB GPU (NVIDIA, Santa Clara, CA, USA). The ECG delineation system was running on Microsoft Windows Server 2019 Standard. All software modules were programmed in Python 3.9.5. Keras and Tensorflow 2.9.1 were used for the neural networks’ implementation, training, and test.

The ECG delineation system could be considered with a hybrid structure, including a deep learning stage (CED-Net, CED-Res-Net, CED-U-Net, or CED-LSTM-Net models) for the segmentation of ECG beats and a machine learning stage (Measurement module) to identify the fiducial points of the waves’ onsets and offsets (Figure 2). Each stage requires computing resources for training and inference. While extensive training resources are required for the initial design time on a PC (all models required from 300 to 400 training epochs), the inference time is finally important for evaluating the real-life applicability. Therefore, we computed the inference time of the machine learning and deep learning stages of the ECG delineation system while running in the development platform. The results were computed for 100 runs of the ECG delineation system on the test dataset, and the inference times of the machine learning and deep learning stages were separately reported as the mean value ± standard deviation (min–max range) for the delineation of one 12-lead representative beat:

- Machine learning (Measurement module): 176 ± 12.3 μs (155–203 μs).
- Deep learning CED-Net: 221 ± 16.3 μs (201–264 μs).
- Deep learning CED-Res-Net: 224 ± 16.5 μs (199–262 μs).
- Deep learning CED-U-Net: 245 ± 16.0 μs (233–287 μs).
- Deep learning CED-LSTM-Net: 430 ± 16.5 μs (408–472 μs).

We observe comparable inference times for CNN-based networks and about twice the runtime of the network with an LSTM layer. Nevertheless, for clinical use, these inference times are acceptable, and even longer execution times can be tolerated in the worst-case scenario when the diagnostic system is not equipped with a GPU.

## 4. Discussion

### 4.1. The Main Findings of This Study

This study presents a comprehensible description of the methodological concept applicable to the training and testing of encoder–decoder DNNs in the 12-lead ECG delineation project related to the diagnostic measurements of P-wave, QRS-, and T-wave boundaries. The main questions typically addressed in the design of such projects are related to the following:

1. The reliability of the reference input and its availability in sufficient amounts of training and test data, ensuring the generalizability of the results.
2. The correctness of the DNN model design and training concept, ensuring the effective training of DNNs to produce outputs that adequately match the reference input.
3. The optimization of DNNs to compare various representative architectures and select the best-performing ones.
4. The validation of the results against independent test datasets and requirements defined in the standards.

The specific decisions involved in this ECG delineation study are discussed in the following subsections, in the context of the questions above and the results achieved.

#### 4.1.1. Training and Test Strategies

Robust deep learning requires substantial amounts of input data that represent normal and abnormal physiological conditions. The training sample size in this study includes 24,232 representative beats with various pathologies found in one of the largest public repositories with clinical 12-lead ECGs, such as the PhysioNet PTB-XL database. This sample size is in the range of the DNN trainable parameters (from about 9000 to 78,000 in Figure 10). Nevertheless, it can be considered sufficient for training small and large models that present comparable validation performances (Figure 11). This is consistent with the results of the study by Götz et al. [88], showing that deep convolutional neural networks can provide equally good classification performance when the training sample size is from 2 times higher down to 10 times lower than the trainable parameters. The generalizability of the best models is justified in inference using data from another sample pool (patients) in the PTB diagnostic database, as well as the CSE database as required in the International standard IEC 60601-2-25:2011 [8].

Given that the PhysioNet PTB-XL and PTB diagnostic databases do not contain delineation information for individual or representative beats, they are not usable in the traditional scenario with human-based supervised learning, which is usually limited to sparse annotated data. Therefore, we introduce the advanced approach for the machine-based supervised learning of DNNs, guided by the diagnostic measurements of an accurate delineation algorithm (ETM) embedded in commercial devices, which is routinely used in clinical practice to support diagnostic decisions made by cardiologists. The reference input is aligned with the traditional diagnostic output of the commercial algorithm, which is provided for representative beats, as they are assumed to be a robust representation of cardiac activity with reduced noise effects. Thus, different DNN architectures can be compared in terms of their capability to reproduce the performance of a commercial ML algorithm. It is worth noting that the presented deep learning strategy is applicable to the delineation of individual beats in case a sufficient amount of reliable reference measurements is available, although noises could have a larger effect on input data. Therefore, the noise robustness of DNN architectures is also a subject of the test results in this study. Lastly and most importantly, we ensure tests to provide compliance with standards, particularly using specific recordings from the CSE database, included in the recommendations for reporting essential performance for ECG interval measurements of diagnostic electrocardiographs.

#### 4.1.2. Model Design and Training

The primary DNN design (CED-Net in Figure 4) is based on the convolutional encoder–decoder concept, which is traditionally effective in image and signal segmentation tasks. Specific to this study is the decision to transform 12-lead ECG input (raw data of representative beat) to three binary outputs, corresponding to the segments of interest (P-wave, QRS-complex, QT-interval). The CED-Net model itself cannot be trained for such binary output transformation but can generate three probabilistic outputs by minimizing a binary cross-entropy loss against binary reference measurements (Figure 2, top). The effective CED-Net training in such a scenario is shown in Figure 7. In the second stage, the interpretation of the CED-Net probabilistic outputs by the threshold-based Measurement module (Figure 2, bottom) is very important to the delineation performance. A comprehensive illustration of the thresholds for P-onset, P-offset, QRS-onset, QRS-offset, and T-offset, which are applied by this module, is shown in Figure 3. The training of the thresholds to provide a zero mean time error (mean(TE)→0) in the training database (Figure 9) is a valid statistical approach to produce outputs that adequately match the reference input. The analysis of the P-wave detection ROC curve and the adjustment of the convex operating point (Figure 8) contribute to establishing a valid P-wave detection threshold.

#### 4.1.3. Model Optimization

In respect to DNN model optimization, basic CED-Net hyperparameters (number of channels and kernel size) are varied (Figure 10), leading to the conclusion that models can be trained with substantially comparable performances (Figure 11). Only one model failed: the one with the channel configuration Ch(6-12-24-24-12-6). This model has the smallest number of channels in the first/last layer of the encoder–decoder, clearly providing insufficient input/output feature space. As can be deduced from Figure 10 and Figure 11, the effective strategies for providing competitive performance by resource-efficient models with a limited number of trainable parameters are as follows: (1) decreasing the number of channels from the bottom to the top of the encoder Ch(24-12-6-6-12-24), presenting a size reduced about 2.9 times in comparison to the fixed channel model Ch({24}); and (2) using a small number of kernels K({4}) as the kernel size linearly determines the model parameters. Nevertheless, our optimization strategy selected the best-performing model (Ch({24}), K({8}), which includes a sufficient number of input channels (doubling the number of input ECG leads to prevent information loss) and a suitably large, but not excessive, kernel size.

The second-level optimization considers variations in CED-Net architectures, including the recurrent LSTM layer (CED-LSTM-Net), residual connections between the encoder–decoder (CED-U-Net), and residual blocks (CED-Res-Net), comprehensively illustrated in Figure 5. Given the different layers and connectivity in the models, comparing their performance with respect to the learning rate (Figure 12) is a valuable monitor of their ability to train effectively. This ensures confidence that the final selection of the best representative for each model is optimally trained. The most robust model is CED-Net, which can find its optimum almost equally effectively at a wide LR range from 0.0001 to 0.01. In contrast, other models with residual connections presented suboptimal training at the lowest LR (0.0001) and the highest LR (≥0.005). The model with an LSTM layer is most affected at a higher LR, failing to train at LR = 0.01, despite several independent training runs. These results indicate that the learning rate is an important hyperparameter for complex neural architectures and should be explored in model optimization, as presented in Figure 12.

#### 4.1.4. Model Test

Evaluating neural networks on an independent test dataset is essential in order to validate their performance, ensure that they generalize well to new data, and build confidence in their practical applicability.

The first part of the test results (Table 1) compares the capability of different DNN encoder–decoders to reproduce the measurements of the reference ML algorithm in the diagnostic PTB database. The test results (Table 1) are very similar to the training results for the highlighted best models (Figure 12), confirming the relatively similar performance of all DNN architectures. All models present P-wave detection performance with the TPR and TNR (94.7–97.9%), corresponding to an average accuracy of 96.5–96.7%, calculated as (TPR + TNR)/2. None of the models are found to fail in the detection of specific fiducial points, providing the most robust detection of the QRS-onset (TE mean value of about 1 ms, standard deviation of ±2.9–5.4 ms) and the least accurate detection of the T-offset (TE standard deviation of ±8.7–9.9 ms) and the P-onset (TE mean value of about 2–3 ms). The durations of the diagnostic intervals are measured within similar error ranges, found with a mean value below 2.5 ms and a standard deviation between 5.9 and 11.4 ms. Based on the test results in Table 1, we can conclude that all DNN architectures are equally capable of reproducing the measurements of the reference ML algorithm for ECG delineation. Without any other evidence of the superiority of a given architecture, we would recommend the lightweight one, which is CED-Net with the fewest parameters.

The second part of the test results (Table 2) validates different DNNs against the standard-based evaluation practices of diagnostic electrocardiographs [8], concerning the accuracy of interval measurements on biological ECGs (P-duration, PQ-interval, QRS-duration, QT-interval). Although in the previous test, DNNs did not show essential differences with the measurements of the reference ETM algorithm embedded in commercial ECG devices, it is important to verify the generalizability of the DNN measurements with the standardized annotations in the CSE database. According to Table 2, all models present a mean duration error in a narrow range (−3.0 to 2.6 ms), which is substantially below the most restrictive standard limit of ±10 ms. The standard deviations of duration errors appear to be related to the type of measured ECG waves, with the lowest deviations observed for the QRS-duration (4.7 to 5.3 ms), well within the ±10 ms limits. For other waves, the deviations are broader, reaching up to about 14 ms. This meets the tolerances for P-duration (±15 ms) and QT-interval (±30 ms) but is critical for the PQ-interval (±10 ms) measured by three of the models (CED-Res-Net, CED-U-Net, and CED-LSTM-Net). Only the CED-Net model with a PQ-interval deviation of 5.8 ms meets all standard tolerances. This is a crucial practical consideration for confident ECG delineation, which can be provided by the basic encoder–decoder architecture embedded in CED-Net.

The third part of the test results (Figure 13) compares the noise immunity of DNNs in noisy conditions, specifically four types of noise: HF, LF, and PL noise at 50 Hz or 60 Hz, added to representative ECGs from the CSE database. The noise tests warn that CED-U-Net measurements might be significantly affected by all types of noise, giving extremity errors (means up to 20 ms, standard deviations up to 65 ms), associated with the following: P-wave prolongation/shortening for HF/LF noise and QRS shortening (LF and PL 60 Hz noise). This is an important finding for the reduced noise immunity of the classical U-net architecture, which is the preferable DNN design in many ECG segmentation studies [56,57,58,59,60,61,62,63,64,65,66]. Although the U-net skip connections have been shown to facilitate training, the direct transfer of information from input to output without passing convolutional filters is unsafe from noise immunity. The other three models (CED-Net, CED-Res-Net, and CED-LSTM-Net) are robust to all types of noise, presenting a mean duration error below ±2.5 ms, which might reach up to +5 ms (prolonged P-duration) for HF and PL noises when compared to measurements without noise. In conclusion, we have found that encoder–decoders with convolutional filters on the signal path and without skip connections that concatenate input to output feature maps provide noise robust ECG delineation. Although these conclusions may not be important for representative beats, they are important for individual beats, where noises could have a larger effect on input data.

### 4.2. Comparative Study with Published ECG Delineation Algorithms

#### 4.2.1. Comparison of P-Wave Detection Performances and Time Errors

**Figure 14:**
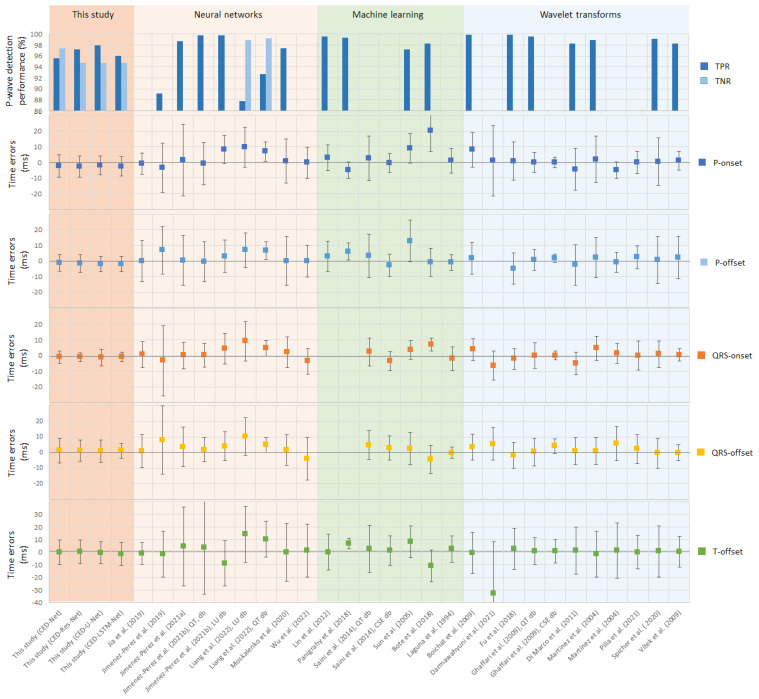
Benchmarking the test performance of this study’s best models (CED-Net, CED-Res-Net, CED-U-Net, and CED-LSTM-Net) against other published studies. Data are presented as percentages for the P-wave detection performance (TPR: true positive rate, TNR: true negative rate) or as mean values (markers) and standard deviations (whiskers) for the time errors for the detection of PQRST fiducial points: P-onset, P-offset, QRS-onset, QRS-offset, and T-offset. Numerical values are disclosed exactly as reported in original articles, where available. Gaps appear where data are not available. Reference studies are grouped (colored) according to the type of their methods employed for ECG delineation: neural networks (Jia et al. (2019) [65], Jimenez-Perez et al. (2019) [60], Jimenez-Perez et al. (2021a) [59], Jimenez-Perez et al. (2021b) [66], Liang et al. (2022) [63], Moskalenko et al. (2020) [58], Wu et al. (2022) [52]), machine learning (Lin et al. (2012) [25], Panigrahy et al. (2018) [24], Saini et al. (2014) [21], Sun et al. (2005) [28], Bote et al. (2018) [16], Laguna et al. (1994) [9]), wavelet transforms (Boichat et al. (2009) [33], Darmawahyuni et al. (2021) [31], Fu et al. 2018 [42], Ghaffari et al. (2009) [43], Di Marco et al. (2011) [32], Martínez et al. (2004) [6], Pilia et al. (2021) [38], Spicher et al. (2020) [34], Vítek et al. (2009) [37]).

The test performances of all models designed in this study (CED-Net, CED-Res-Net, CED-U-Net, and CED-LSTM-Net), as presented in Table 1, have been compared to the results reported in other published studies on ECG delineation. The comparative study is graphically summarized in Figure 14, presenting the P-wave detection performance (TPR, TNR) on top and the time errors (P-onset, P-offset, QRS-onset, QRS-offset, T-offset) on the bottom. The numerical values are reported as read from original articles (mean ± standard deviation, or percentages). The studies are grouped by color according to the applied methods, involving NNs or ML or WT algorithms. 

It is worth mentioning that the comparisons in Figure 14 have been made under different conditions in respect to the databases used for reporting the results in published studies. Published NN studies used either five-fold cross-validation [52,59,65], the subject-wise splitting of a single database [58,60,63], or testing with an independent database [66] as in this study. In contrast, WT [6,31,32,33,34,37,38,43] and ML algorithms [9,16,24,25,28] did not address training data but just reported validation results on the database involved in the development process, except for WT [42] and ML [22]. The differences also concern the input data, specifically the number of ECG leads and the type of beats analyzed. This study uses 12 leads, whereas most other studies use only 2 leads. Additionally, this study focuses on representative beats, in contrast to the individual beats analyzed in other studies.

The P-wave detection performance is commonly reported by the metric TPR, which varies across studies: 89.1–99.8% for NNs, 97.2–99.5% for ML, and 98.2–99.9% for WT. The present study reports a TPR of 95.6–97.9%, falling within the range of other NNs. Since the TPR reflects the rate of false negatives, it is statistically appropriate to also report the TNR, which indicates the rate of false positives. In this study, the TNR ranges from 94.7 to 97.4%, similar to the TPR range, providing a balanced detection of the P-wave when present or absent. The average accuracy, calculated as (TPR + TNR)/2, is 96.5–96.7%. This study demonstrates balanced accuracy for P-wave detection unlike [63] with a TNR up to 10% higher than the TPR (98.9–99.2% vs. 87.7–92.7%). Unfortunately, other published studies have not reported the TNR at all.

The time errors in Figure 14 can be easily interpreted: more accurate methods can be recognized by markers near zero for the mean TE and narrower whiskers representing the TE standard deviation. This study appears with mean markers in the range (−2.5 to 1.3 ms) and standard deviation whiskers in the range (2.9 to 9.9 ms) for all ECG fiducial points. This performance can be recognized in the lower range of time errors, comparable to the following studies:

- NN studies: P-onset [63], QRS-offset [63,66], and T-offset [65];
- ML studies: P-onset [9,23], P-offset [9], QRS-offset [6,9,23], and T-offset [9,23,24];
- WT studies: P-onset [37,38,43], P-offset [43], QRS-onset [37,43], QRS-offset [32,34,37,42,43], and T-offset [43].

The remaining studies report a higher mean time error and/or wider standard deviations.

#### 4.2.2. Comparison of Duration Errors Calculated According to Standard-Based Evaluation Practice

We have found only two studies [22,89] reporting duration errors of diagnostic ECG intervals according to the standard-based evaluation practice using the CSE dataset, to which this study is compared in Table 3.

- P-duration: This study exhibits one of the lowest mean P-duration errors and standard deviations similar to other studies, all within standard tolerance [8], except CSE Prog. 2 (Marquette) [89].
- PQ-interval: The standard tolerance [8] is fulfilled only by four methods, including CED-Net (this study), KNN and SVN [22], and CSE Prog. 13 (Padova) [89]. The ±10 ms standard deviation tolerance is problematic for the other five methods, which exceed this limit by 2 to 4 ms.
- QRS-duration: All studies are well below the standard tolerance of ±10 ms [8], with a mean value of ±(0.8–3 ms) and standard deviation ±(4.7–7.3 ms).
- QT-interval: The standard tolerances [8] are wider, allowing for larger errors in the T-offset localization. Nevertheless, all studies do not present important problems with QT-interval measurements, having the following: mean errors within ±2 ms (this study), ±5 ms [22] and ±6.2 ms [89], compared to the ±25 ms standard tolerance; and standard deviations within ±15.4 ms compared to the ±30 ms standard tolerance.

In conclusion, full compliance with standard tolerances [8] can be highlighted for CED-Net (this study), KNN and SVN [22], and CSE Prog. 13 (Padova) [89].

## 5. Conclusions

This study investigates DNNs, which transform 12-lead representative beats to three diagnostic ECG intervals (P-wave, QRS-complex, and QT-interval) used for the global delineation of the representative beat (P-onset, P-offset, QRS-onset, QRS-offset, and T-offset). Although neural network-based models for ECG time series analysis are not a new frontier, to the best of our knowledge, this is the first study to directly compare the ECG delineation performances of different encoder–decoder architectures applied on clinical and standard ECG databases and noise tests. Four concepts for encoder–decoders based on a fully convolutional architecture (CED-Net) and its modifications with a recurrent layer (CED-LSTM-Net), residual connections between symmetrical encoder and decoder feature maps (CED-U-Net), and sequential residual blocks (CED-Res-Net) are involved in a fair comparison. All encoder–decoders were trained and optimized with the large PhysioNet ECG database (PTB-XL) under identical conditions, applying an advanced approach for machine-based supervised learning with a commercial ML algorithm for ECG delineation. The test results demonstrate the equal capability of all DNN architectures to reproduce the measurements of the reference ML algorithm in the diagnostic PTB database with an average P-wave detection accuracy (96.6%), time and duration error means (−2.6 to 2.4 ms), and standard deviations (2.9 to 11.4 ms). The validation according to the standard-based evaluation practices of diagnostic electrocardiographs with the CSE database outlines the CED-Net model, which measures P-duration (2.6 ± 11.0 ms), PQ-interval (0.9 ± 5.8 ms), QRS-duration (−2.4 ± 5.4 ms), and QT-interval (−0.7 ± 10.3 ms), which meet all standard tolerances. Noise tests with HF, LF, and PL noise at 50 Hz or 60 Hz confirm that CED-Net, CED-Res-Net, and CED-LSTM-Net are robust to all types of noise, mostly presenting a mean duration error below ±2.5 ms when compared to measurements without noise. Reduced noise immunity is observed for the U-net architecture. Comparative analysis with other published studies scores this research within the lower range of time errors, highlighting its competitive performance.

In this study, we have looked at the neural networks applied to the delineation of representative beats. Future research could consider another sample pool aside from the PhysioNet ECG database, data augmentation with noise components, a detailed ECG delineation of the QRS-complex, ST-segment, and T-wave, and the delineation of multiple PQRST segments in the ECG recording rather than representative beats.

## Figures and Tables

**Table 1:** Test performance of CED-Net, CED-Res-Net, CED-U-Net, and CED-LSTM-Net models with PTB diagnostic database. Categorical data for P-wave detection performance are presented as percentage (number). Continuous data for time and duration errors are presented as mean value (±standard deviation) in non-outlier range.

PTB Diagnostic Database	CED-Net	CED-Res-Net	CED-U-Net	CED-LSTM-Net
**P-wave detection**				
TPR, % (number)	95.6% (504/527)	97.2% (512/527)	97.9% (516/527)	96.0% (506/527)
TNR, % (number)	97.4% (74/76)	94.7% (72/76)	94.7% (72/76)	94.7% (72/76)
**Time Errors**				
P-onset, ms	−2.3 (±7.3)	−2.6 (±6.7)	−1.9 (±6.0)	−2.5 (±6.2)
P-offset, ms	−1.4 (±5.2)	−1.7 (±5.5)	−2.0 (±5.0)	−1.9 (±4.9)
QRS-onset, ms	−1.1 (±3.8)	−1.1 (±2.9)	−1.3 (±5.4)	−0.9 (±3.0)
QRS-offset, ms	1.3 (±7.9)	1.1 (±6.7)	0.8 (±7.2)	1.1 (±4.7)
T-offset, ms	−0.1 (±9.9)	0.3 (±9.3)	−0.5 (±8.7)	−1.4 (±9.1)
**Duration Errors**				
P-duration, ms	1.0 (±9.3)	0.9 (±8.5)	−0.2 (±8.4)	0.7 (±8.3)
PQ-interval, ms	1.3 (±7.5)	1.7 (±6.8)	0.9 (±6.6)	1.7 (±6.6)
QRS-duration, ms	2.4 (±9.9)	2.3 (±7.4)	2.1 (±10.6)	2.0 (±5.9)
QT-interval, ms	1.0 (±11.2)	1.4 (±9.9)	0.6 (±11.4)	−0.4 (±9.9)

**Table 2:** Test performance according to standard-based evaluation practice with CSE dataset. Data are presented as mean value (± standard deviation).

Duration Errors	Standard Tolerances [8]	CED-Net	CED-Res-Net	CED-U-Net	CED-LSTM-Net
P-duration, ms	±10 (±15)	2.6 (±11.0)	−0.6 (±11.5)	−0.4 (±12.9)	−0.5 (±11.4)
PQ-interval, ms	±10 (±10)	0.9 (±5.8)	−0.8 (±14.0 *)	−0.7 (±13.8 *)	−0.3 (±13.7 *)
QRS-duration, ms	±10 (±10)	−2.4 (±5.4)	−2.3 (±5.3)	−3.0 (±4.7)	−2.1 (±5.3)
QT-interval, ms	±25 (±30)	−0.7 (±10.3)	−0.9 (±9.1)	−0.6 (±11.0)	−1.9 (±10.2)

* Value not compliant with the standard tolerance.

**Table 3:** Benchmarking the duration errors of this study’s best models (CED-Net, CED-Res-Net, CED-U-Net, and CED-LSTM-Net) against other published studies according to the standard-based evaluation practice with the CSE dataset. Data are presented as the mean value (±standard deviation).

Study	Method	P-Duration (ms)	PQ-Interval (ms)	QRS-Duration (ms)	QT-Interval (ms)
**[8]**	**Standard Tolerances**	**±10 (±15)**	**±10 (±10)**	**±10 (±10)**	**±25 (±30)**
This study	CED-Net	2.6 (±11.0)	0.9 (±5.8)	−2.4 (±5.4)	−0.7 (±10.3)
This study	CED-Res-Net	−0.6 (±11.5)	−0.8 (±14.0 *)	−2.3 (±5.3)	−0.9 (±9.1)
This study	CED-U-Net	−0.4 (±12.9)	−0.7 (±13.8 *)	−3.0 (±4.7)	−0.6 (±11.0)
This study	CED-LSTM-Net	−0.5 (±11.4)	−0.3 (±13.7 *)	−2.1 (±5.3)	−1.9 (±10.2)
[22]	KNN	−2.5 (±9.3)	5.4 (±9.9)	−2.3 (±7.2)	5.0 (±13.4)
[22]	SVN	−0.1 (±7.9)	2.3 (±7.1)	−1.1 (±7.0)	3.9 (±11.8)
[89]	CSE Prog. 2 (Marquette)	−12.0 * (±17.6 *)	−8.7 (±12.1 *)	−0.8 (±7.2)	6.2 (±15.4)
[89]	CSE Prog. 11 (Glasgow)	−2.4 (±12.6)	5.1 (±12.5 *)	−1.4 (±7.1)	3.9 (±14.8)
[89]	CSE Prog. 13 (Padova)	2.8 (±10.1)	−2.8 (±8.3)	1.8 (±7.3)	−1.1 (±9.2)

* Value not compliant with the standard tolerance.

## Data Availability

The PTB-XL dataset is publicly available through the PhysioNet website at https://physionet.org/content/ptb-xl/1.0.1/, last accessed on 7 June 2024. Restrictions apply to the availability of the test CTS dataset [84], on which the authors are not permitted to release the digital data.
